# Reduction Mammoplasty: A Comparison Between Operations Performed by Plastic Surgery and General Surgery

**Published:** 2015-09-22

**Authors:** Anthony M. Kordahi, Ian C. Hoppe, Edward S. Lee

**Affiliations:** ^a^Rutgers–New Jersey Medical School, Newark; ^b^Department of Surgery, Division of Plastic Surgery, Rutgers–New Jersey Medical School, Newark

**Keywords:** reduction mammoplasty, quality improvement, plastic surgery, general surgery, outcome assessment (health care)

## Abstract

**Background:** Reduction mammoplasty is an often-performed procedure by plastic surgeons and increasingly by general surgeons. The question has been posed in both general surgical literature and plastic surgical literature as to whether this procedure should remain the domain of surgical specialists. Some general surgeons are trained in breast reductions, whereas all plastic surgeons receive training in this procedure. The National Surgical Quality Improvement Project provides a unique opportunity to compare the 2 surgical specialties in an unbiased manner in terms of preoperative comorbidities and 30-day postoperative complications. **Methods:** The National Surgical Quality Improvement Project database was queried for the years 2005–2012. Patients were identified as having undergone a reduction mammoplasty by *Current Procedural Terminology* codes. Results were refined to include only females with an *International Classification of Diseases, Ninth Revision*, code of 611.1 (hypertrophy of breasts). Information was collected regarding age, surgical specialty performing procedure, body mass index, and other preoperative variables. The outcomes utilized were presence of superficial surgical site infection, presence of deep surgical site infection, presence of wound dehiscence, postoperative respiratory compromise, pulmonary embolism, deep vein thrombosis, perioperative transfusion, operative time, reintubation, reoperation, and length of hospital stay. **Results**: During this time period, there were 6239 reduction mammaplasties performed within the National Surgical Quality Improvement Project database: 339 by general surgery and 5900 by plastic surgery. No statistical differences were detected between the 2 groups with regard to superficial wound infections, deep wound infections, organ space infections, or wound dehiscence. There were no significant differences noted between within groups with regard to systemic postoperative complications. Patients undergoing a procedure by general surgery were more likely to experience a failure of skin flaps, necessitating a return to the operative room (*P* < .05). Operative time was longer in procedures performed by general surgery (*P* < .05). **Conclusion:** Several important differences appear to exist between reduction mammaplasties performed by general surgery and plastic surgery. A focused training in reduction mammoplasty appears to be beneficial to the patient. The limitations of this study include a lack of long-term follow-up with regard to aesthetic outcome, nipple malposition, nipple sensation, and late wound sequelae.

Reduction mammoplasty is a commonly performed procedure with the goals of reducing the weight and volume of the breast. The indications for the procedure include both physical and psychological concerns. The chronic weight and pressure placed on brassiere straps cause deep grooves to form in the trapezius muscles, there may be back and chest wall pain, and the inframammary region becomes subject to rashes and maceration. Gigantomastia can be an embarrassing focus of attention for women of all ages, especially if unilateral hypertrophy with asymmetry exists. Moreover, excessively large breast size may hinder women from being able to participate in forms of exercise or other activities of daily functioning, which can lead to future health concerns.^1^

The earliest recorded reduction mammoplasty procedures were documented in the late 1800s with techniques developed by surgeons who were not concerned with the aesthetic outcome.^2^ Over the years, techniques have been developed and refined to achieve both aesthetic and functional results. No single technique can be applied to all breasts, and surgeons must adapt their procedure to accommodate the patients’ unique presentations.

While there are some general surgeons who receive training in reduction mammoplasty, oftentimes through an oncoplastic breast fellowship, all plastic surgeons are trained in this procedure. In the past, there have been several studies in the literature comparing the outcomes of reduction mammoplasty between plastic surgeons and general surgeons.^3^^,^^4^ One suggested that there is no difference between plastic surgeons and general surgeons performing the procedure.^3^ However, most of these studies involved a low number of cases and subjective questionnaires determining patients’ assessment of their results. Whether this procedure ought to solely remain in the realm of surgical specialists has a large impact on both surgical training and patient outcomes.^4^

As the number of reduction mammoplasty procedures in the United States increases, the demand for surgeons and their time does as well. According to statistics posted on the Association of American Medical Colleges Web site, the number of plastic surgeons per 100,000 population has decreased from 2.21 to 2.11 from 2006 to 2013.^5^ One study demonstrated that geographic accessibility to a plastic surgeon is an important factor in determining whether or not a patient will undergo a reduction mammoplasty procedure.^6^ These factors are likely increasing the impetus for general surgeons to begin performing this procedure.

The goal of this study is to determine whether or not reduction mammoplasty has similar outcomes when performed by general surgeons as compared with plastic surgeons. To accomplish this objective, information provided by the American College of Surgeons National Surgical Quality Improvement Program (NSQIP) database was used. NSQIP began as a program mandated by Congress in 1988 to assess the morbidity and mortality rates of surgical cases in Veteran Affairs hospitals.^7^ Since then, it has evolved into a vast, international database that collects clinical data points from more than 250 participating hospitals for up to 30 days postoperatively. It also analyzes, organizes, and provides these data for the benefit of the participating hospitals with the purpose of increasing the standard of surgical quality.^8^

## METHODS

Following institutional review board approval, the participant use files from the NSQIP database were analyzed for the years 2005–2012. Patients who had undergone a reduction mammoplasty were identified by *Current Procedural Terminology* codes, and these results were then refined to include only females with a *International Classification of Diseases*, *Ninth Revision*, code of 611.1 (hypertrophy of breasts). The database provided patient information including general demographics, surgical specialty performing the procedure, as well as other preoperative variables. To compare outcomes of the procedures, the variables utilized included presence of superficial surgical site infection, presence of deep surgical site infection, presence of wound dehiscence, postoperative respiratory compromise, pulmonary embolism, deep vein thrombosis, perioperative transfusion, operative time, reintubation, reoperation, and length of hospital stay. Chi-squared and *t*-test statistical analyses were utilized with a significance level of 5%.

## RESULTS

**Figure 1:**
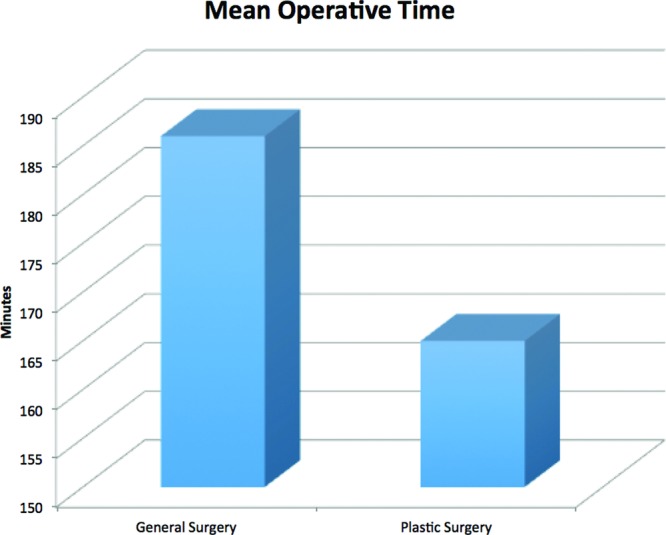
Mean operative time.

During the time period, a total of 6239 reduction mammoplasties were performed and recorded within the NSQIP database, of which 339 were performed by general surgeons and 5900 by plastic surgeons. Table 1 describes the general demographics and preoperative variables analyzed for the patient cohort. Patients operated by general surgery tended to be younger with a greater body mass index. Postoperative analysis depicts that there were no statistically significant differences between the 2 groups with regard to superficial wound infections, deep wound infections, organ space infections, wound dehiscence, or systemic postoperative complications. This information is depicted in Table 2. However, there was a significant difference (*P* < .05) calculated in the number of patients experiencing a failure of skin flaps, which necessitated a return to the operating room. The mean operative time was longer for general surgeons (186 minutes) than for plastic surgeons (165 minutes), as depicted in Figure 1.

## DISCUSSION

Analysis of the results indicates that general surgeons take a longer time to perform a reduction mammoplasty and may be prone to an increased rate of complications. During general surgery residency, exposure to procedures outside of the standard curriculum may vary between institutions. In comparison, reduction mammoplasty is a procedure that is required by the plastic surgery resident review committee. One study applied a unique set of quality and efficiency metrics, including a consideration of the learning curve for given procedures, elucidation of factors that influence operative performance, procedure-specific complications, and definitions of acceptable variance ranges from established norms for reduction mammoplasty.^9^ This study suggested a 3-phase learning curve for reduction mammoplasty procedures in which complication rates, variance in operative time, and operative time all are inversely correlated with surgeons’ experience. Most importantly, operative efficiency and quality did not reach a phase of stabilization until approximately the 12th year of operative experience.^9^ While plastic surgeons must be specifically trained in reduction mammoplasty, numerous other procedures performed on the breast provide a more comprehensive understanding of the aesthetic concerns specific for this anatomic area.

Other variables not assessed in the NSQIP database may also lead to greater patient success with a plastic surgeon. Functionally speaking, a decrease in breast mass and volume will achieve the basic goals of a reduction mammoplasty; however, there still remains subjective measures of outcomes including aesthetics as well as nipple and areola sensitivity. With regard to postsurgical breast sensitivity, studies have shown that there exists an inverse relationship between breast size and sensitivity, as well as an improvement in sensitivity after breast reduction.^10^

While most insurance carriers will not reimburse for procedures involving less than 1000 g, studies have shown that reduction mammoplasties involving less than 1000 g offer substantial relief of macromastia-associated symptoms and significant improvement in the patients’ quality of life.^11^ It is clear that economic constraints and an increasing demand for reduction mammoplasty have placed a premium on increased efficiency and optimization of techniques. The data presented earlier support that the knowledge of various surgical techniques and comfort with the procedure lead to the efficient use of surgeon and operating room resources as well as optimal outcomes for patients.^12^

## CONCLUSION

The focused training in reduction mammoplasty that plastic surgeons receive appears to benefit the patient. These results may be further emphasized with the increasing number of patients desiring reduction mammoplasty procedure due to knowledge and availability. The limitations of this study include an inability to monitor patient outcomes past the 30 day postoperative period, relatively low number of procedures performed by general surgery, and absence of variables such as nipple malposition, nipple sensation, aesthetic outcome, and late wound sequelae.

## Figures and Tables

**Table 1:** Preoperative characteristics*

	General surgery	Plastic surgery	*P*
*n*	339	5900	
Mean age	40.9	43	<.01
Mean body mass index	33	31.3	<.001
Diabetes	16 (4.7%)	276 (4.7%)	.97
Current smoker	30 (8.9%)	664 (11.3%)	.17
Recent alcohol use	3 (1.2%)	26 (0.7%)	.32
Dyspnea	14 (4.1%)	151 (2.6%)	.08
COPD	3 (0.9%)	46 (0.8%)	.83
CHF	0 (0%)	2 (0.03%)	.73
Myocardial infarction	0 (0%)	0 (0%)	
Previous PCI	0 (0%)	24 (0.6%)	.21
Previous cardiac surgery	1 (0.4%)	18 (0.5%)	.89
Angina	1 (0.4%)	2 (0.1%)	.05
Hypertension	85 (25.1%)	1313 (22.3%)	.23
PVD	0 (0%)	2 (0.1%)	.72
Preoperative wound infection	1 (0.3%)	14 (0.2%)	.83
Chronic steroid use	2 (0.6%)	63 (1.1%)	.4
Bleeding disorder	0 (0%)	30 (0.5%)	.19

*COPD indicates chronic obstructive pulmonary disease; CHF, congestive heart failure; PCI, percutaneous coronary intervention; PVD, peripheral vascular disease.

**Table 2:** Postoperative complications*

	General surgery	Plastic surgery	*P*
Superficial wound infection	9 (2.7%)	179 (3.0%)	.69
Deep wound infection	0 (0%)	29 (0.5%)	.2
Organ space infection	0 (0%)	4 (0.1%)	.63
Wound dehiscence	5 (1.5%)	40 (0.7%)	.09
Pneumonia	0 (0%)	1 (0.002%)	.81
Reintubation	0 (0%)	3 (0.1%)	.68
Pulmonary embolism	0 (0%)	10 (0.2%)	.45
On ventilator >48 h	0 (0%)	2 (0.03%)	.74
Renal insufficiency	0 (0%)	0 (0%)	
Renal failure	0 (0%)	1 (0.002%)	.81
Urinary tract infection	1 (0.3%)	4 (0.1%)	.15
CVA	0 (0%)	2 (0.03%)	.74
Coma	0 (0%)	0 (0%)	
Neurological deficit	0 (0%)	0 (0%)	
Cardiac arrest	0 (0%)	0 (0%)	
Myocardial infarction	0 (0%)	0 (0%)	
Blood transfusion	0 (0%)	20 (0.3%)	.28
Failure of graft, myocutaneous flaps, skin graft requiring return to OR	1 (0.3%)	2 (0.03%)	.03
DVT	0 (0%)	4 (0.1%)	.63
Sepsis	0 (0%)	5 (0.1%)	.59
Septic shock	0 (0%)	0 (0%)	
Return to OR	6 (1.8%)	104 (1.8%)	.99

*CVA indicates cardiovascular accident; DVT, deep vein thrombosis; OR, operating room.
